# Association of Early Beta Human Chorionic Gonadotropin With Ischemic Placental Disease in Singleton Pregnancies After In Vitro Fertilization

**DOI:** 10.7759/cureus.28117

**Published:** 2022-08-17

**Authors:** Jaimin S Shah, Anna M Modest, Michele R Hacker, Nina Resetkova, Laura E Dodge

**Affiliations:** 1 Obstetrics and Gynecology, Beth Israel Deaconess Medical Center, Harvard Medical School, Boston, USA; 2 Reproductive Endocrinology and Infertility, Boston In Vitro Fertilization (IVF), Waltham, USA; 3 Obstetrics, Gynecology and Reproductive Biology, Harvard Medical School, Boston, USA; 4 Epidemiology, Harvard T.H. Chan School of Public Health, Boston, USA

**Keywords:** small for gestational age, preeclampsia, placental abruption, ischemic placental disease, in vitro fertilization, beta human chorionic gonadotropin

## Abstract

Objectives: To evaluate whether an initial or two-day percent increase in serum beta-human chorionic gonadotropin (βhCG) is associated with ischemic placental disease (IPD) in singleton pregnancies after autologous or donor IVF.

Study design: This was a secondary analysis of a retrospective cohort study of deliveries linked to IVF cycles at a single academic tertiary hospital and infertility treatment center. We included all patients (≥18 years old) who had a singleton live birth or intrauterine fetal demise (IUFD) resulting from either autologous fresh (n=1,347), autologous frozen (n=454), or donor (n=253) IVF cycles.

Main outcome reassures: The primary outcome was a composite outcome of IPD or IUFD due to placental insufficiency. IPDs included preeclampsia, placental abruption, and small for gestational age (SGA).

Results: Neither initial βhCG nor two-day percent increases in βhCG were associated with an increased risk of IPD for any type of IVF cycle. Initial and two-day percent increases in βhCG were significantly higher when comparing frozen with fresh IVF and donor with autologous IVF (all P≤0.01).

Conclusions: Among singleton autologous and donor IVF cycles, the initial and two-day percent increase in serum βhCG were not associated with IPD or its components. However, significant βhCG differences existed by cycle type and oocyte source.

## Introduction

The process by which placentation occurs early in pregnancy plays an important role in perinatal outcomes [1]. Ischemic placental disease (IPD) includes preeclampsia, placental abruption, and small for gestational age (SGA), all of which share the underlying mechanism of uteroplacental insufficiency [2]. Uncertainty regarding the exact cause of IPD remains, but it is believed to stem from abnormal trophoblast invasion that impedes normal spiral artery development, with the severity of the disease depending on the extent of invasion [2-4].

In vitro fertilization (IVF) is associated with an increased risk of IPD and its components [5], with evidence of lower IPD risk in frozen IVF cycles compared with fresh IVF cycles [5]. This difference in risk may be due to differences in ovarian stimulation between fresh and frozen cycles, which could alter angiogenesis and lead to placental insufficiency [6,7]. Furthermore, the oocyte source is associated with IPD risk [8], possibly due to differences in maternal immune tolerance [4].

Early pregnancy is monitored with the use of beta-human chorionic gonadotropin (βhCG), which can serve as a marker of placental development [9]. Previous studies have demonstrated the value of βhCG rise in predicting ongoing pregnancy in both spontaneous conception and IVF [10,11]. Furthermore, studies have shown early βhCG to help predict pregnancy viability [12], duration of pregnancy [13], clinical pregnancy rates [14], and delivery rates [15]. However, data on the initial or two-day percent increase in βhCG in association with perinatal outcomes is lacking. Specifically, there is a paucity of data on the association of early βhCG from IVF pregnancies with IPD and its components.

Ryniec and Esfandiari reviewed early βhCG and its utility within IVF, as there are many factors during the IVF process that could alter the initial levels and kinetics of βhCG compared to spontaneous conception. They concluded that information gathered from early markers in the IVF process can provide a better understanding of pregnancy potential, allow for earlier diagnosis of abnormal pregnancy, and aid in patient counseling [16]. The purpose of this study was to determine whether associations exist between initial or two-day percent increase in βhCG among singleton deliveries resulting from autologous or donor IVF. A better understanding of these associations could be useful for optimizing outcomes for IVF pregnancies. This article was previously presented as a meeting abstract at the 2020 American Society for Reproductive Medicine meeting on October 17, 2020, which was conducted virtually.

## Materials and methods

This was a secondary analysis of an existing retrospective cohort study. The methods for this cohort and linkage have been described previously [5,8]. Briefly, deliveries at a tertiary care hospital from January 1, 2000 to August 31, 2018 were linked to IVF cycles performed by the Division of Reproductive Endocrinology and Infertility. In addition, deliveries were linked to birth certificates from the Massachusetts Department of Public Health. All deliveries were ≥20 weeks of gestation. For this analysis, we included patients at least 18 years old who had a singleton live birth or intrauterine fetal demise (IUFD) resulting from autologous (fresh or frozen) or donor IVF performed at our IVF center. We obtained institutional review board approval at our institution and from the Massachusetts Department of Public Health (protocol #2019P-000212).

Deliveries were included if at least one serum βhCG was recorded during the IVF cycle. The two-day percent increase was extrapolated for cycles with βhCG measured 1, 3, or 4 days after the initial measurement. Serum βhCG concentrations were measured with the immunoassay on the Siemens Immulite 2000 platform prior to July 2012 and the Roche Cobas e411 platform currently. Internal validation and comparison studies were performed when the platform changed per the College of American Pathologists regulations to ensure valid and accurate results. βhCG results were reported in mIU/mL, using the third international reference βhCG standard. Baseline demographic characteristics, obstetrical and infertility history, and delivery outcomes were obtained from electronic medical records. Additional baseline demographic characteristics were obtained from the birth certificate data.

All patients underwent standard ovarian stimulation protocols; blood and ultrasound monitoring; oocyte retrievals; and embryo transfer. Patients either had a cleavage stage or blastocyst embryo transfer in an autologous or donor IVF cycle. For donor IVF cycles, donated oocytes were most frequently obtained from a frozen oocyte bank, with the occasional fresh oocyte donation. The selection of embryo transfer is based on our center’s standard transfer protocol, which incorporates the Gardner morphology [17] and embryo day. The number of embryos transferred was in accordance with American Society for Reproductive Medicine guidelines [18], with possible variations determined by the doctor of record. Supernumerary embryos were cryopreserved at the cleavage or blastocyst stage by slow cooling until August 2011. After that, vitrification, or rapid cooling, was introduced, and our center completely transitioned to vitrification of blastocysts by July 2013. For frozen IVF cycles, patients either underwent an exogenous estrogen and progesterone protocol or a natural thaw cycle per our center’s standard practice.

The primary outcome was IPD (IUFD due to placental insufficiency; preeclampsia; placental abruption; and/or SGA). IPD and its components were verified from medical records according to the International Classification of Diseases, Ninth Revision (ICD-9) and Tenth Revision (ICD-10) codes as described previously [8]. Preeclampsia was defined as the presence of elevated blood pressure (≥140/90) during the admission for delivery and either clinical symptoms of preeclampsia (headache, visual changes, and/or severe right upper abdominal pain), seizures, or abnormal laboratory values (proteinuria, alanine aminotransferase/aspartate aminotransferase ≥80 units per liter, and/or platelets <100,000). SGA was defined as birthweight <10th percentile by neonatal sex and gestational age at delivery [19]. Cases of IUFD were included if they were attributable to placental insufficiency; to make this determination, autopsy, pathology, and clinical notes were reviewed.

For autologous cycles, we categorized initial βhCG in increments of 100 mIU/ml up to 700 mIU/ml; due to fewer donor cycles, we categorized initial βhCG in increments of 150 mIU/ml up to 450 mIU/ml among these cycles. Data are presented as mean ± standard deviation or median (interquartile range). We compared means, medians, and proportions using a t, Wilcoxon, or chi-square test. We used log-binomial regression to estimate risk ratios (RR) and 95% confidence intervals (CI) for the risk of IPD and its components among first deliveries at our institution, adjusting for maternal age and parity, as well as embryo age at the time of initial βhCG measurement for models of initial βhCG. All tests were two-sided, and P-values <0.05 were considered to be statistically significant. Analyses were completed using SAS 9.4 (SAS Institute, Cary, NC) and GraphPad Prism (GraphPad Prism for Windows, GraphPad Software, San Diego, CA).

## Results

Of 2,213 IVF cycles linked to singleton deliveries, 158 (7%) were excluded; 63 (3%) had no initial βhCG measurement, and 96 (4%) had βhCG measured at a time outside the study period. The final sample size of 2,054 IVF cycles included autologous fresh (n=1,347), autologous frozen (n=454), and donor (n=253) IVF cycles. Patient demographics and clinical characteristics are shown in Table 1. Donor IVF patients were older than those undergoing autologous IVF; all groups were predominately white, married/partnered, and college-educated.

**Table 1:** Baseline patient characteristics of cycles with at least one measured βhCG resulting in a singleton delivery by mode of conception (n=2,054 cycles; n=1,821 people)

Characteristic	Autologous Fresh IVF (n=1,347)	Autologous Frozen IVF (n=454)	Donor IVF (n=253)
Maternal age (years)	36.1 (33.2–38.9)	35.8 (33.1–38.5)	42.8 (40.1–45.3)
Race			
White	1,093 (81.1)	322 (70.9)	202 (79.8)
Asian	106 (7.8)	46 (10.1)	16 (6.3)
Black/African American	49 (3.6)	19 (4.2)	9 (3.6)
Hispanic	26 (1.9)	11 (2.4)	NR
Other	55 (4.1)	28 (6.2)	14 (5.5)
Not reported/unknown	18 (1.3)	28 (6.2)	NR
Married or partnered	1,254 (93.1)	404 (89.0)	212 (83.8)
The highest level of education achieved			
College/associate’s degree or less	656 (48.7)	66 (15.4)	84 (33.2)
Graduate degree	504 (37.4)	75 (16.5)	86 (34.0)
Unknown	187 (13.9)	313 (68.9)	83 (32.8)
Gravidity			
1	900 (66.8)	196 (43.2)	136 (53.8)
2	232 (17.2)	135 (29.7)	60 (23.7)
3+	213 (15.8)	123 (27.1)	57 (22.5)
Missing	2 (0.2)	0 (0.0)	0 (0.0)
Parity			
1	1,274 (94.6)	417 (91.9)	233 (92.1)
2+	71 (5.3)	37 (8.2)	20 (7.9)
Missing	2 (0.2)	0 (0.0)	0 (0.0)
Year of delivery			
2000-2005	454 (33.7)	3 (0.7)	56 (22.1)
2006-2011	478 (35.5)	70 (15.4)	72 (28.5)
2012-2018	415 (30.8)	381 (83.9)	125 (49.4)
Infertility diagnosis*			
Male factor	294 (21.8)	35 (7.7)	9 (3.6)
Ovarian dysfunction	133 (9.9)	17 (3.7)	15 (5.9)
Tubal factor	104 (7.7)	11 (2.4)	4 (1.6)
Diminished ovarian reserve	65 (4.8)	6 (1.3)	82 (32.4)
Endometriosis	65 (4.8)	6 (1.3)	3 (1.2)
Uterine	34 (2.5)	5 (1.1)	0 (0.0)
Unexplained	426 (31.6)	53 (11.7)	6 (2.4)
Missing	151 (11.2)	304 (67.0)	76 (30.0)
Peak serum E2 (pg/mL)	1,327 (926–1,976)	--	--
No. of embryos transferred	2.0 (2.0–3.0)	1.0 (1.0–2.0)	2.0 (1.0–2.0)

Pregnancy and delivery outcomes among all IVF cycles are shown in Supplemental Table 1. The gestational age at delivery, incidence of preterm birth, and neonatal sex were similar across all groups. Birthweights were similar for donor and autologous fresh IVF, while autologous frozen IVF was associated with a borderline-significantly higher birthweight compared with donor IVF (P=0.06). Day of embryo transfer differed significantly between donor and autologous fresh IVF and donor and autologous frozen IVF (both P<0.001).

**Figure 1:**
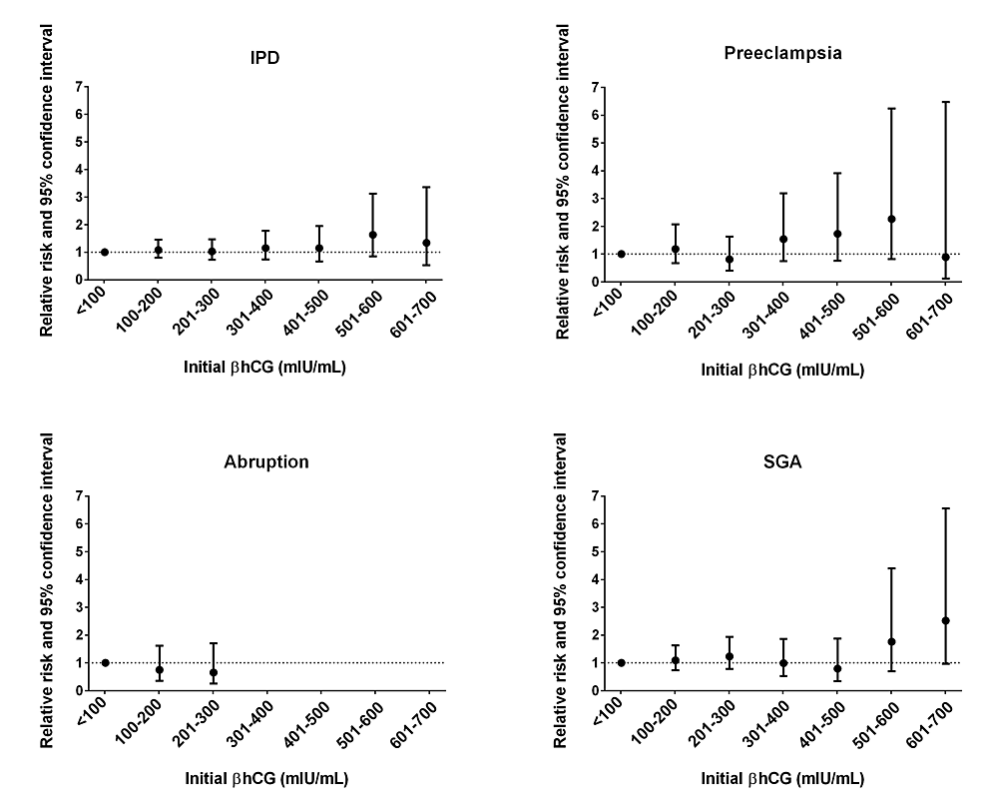
Adjusted risk ratios of ischemic placental disease and its components in first autologous IVF singleton pregnancies by thresholds of initial βhCG Note: Data presented as risk ratio and 95% CI. Adjusted for maternal age, parity, and embryo age at time of initial βhCG with the exception of donor preeclampsia, which was adjusted for maternal age and parity due to convergence issues. IPD: ischemic placental disease; SGA: small for gestational age. 0-100 (n=349), 101-200 (n=620), 201-300 (n=325), 301-400 (n=151), 401-500 (n=89), 501-600 (n=36), 601-700 (n=19).

Among all cycles, there was no difference in mean initial βhCG between pregnancies with (208 mIU/mL, 95% CI: 202-215) and without IPD (209 mIU/mL, 95% 195-224) (P=0.90). We did not observe any significant associations between thresholds of initial βhCG and IPD or its components among patients from autologous IVF cycles (Figure 1).

**Figure 2:**
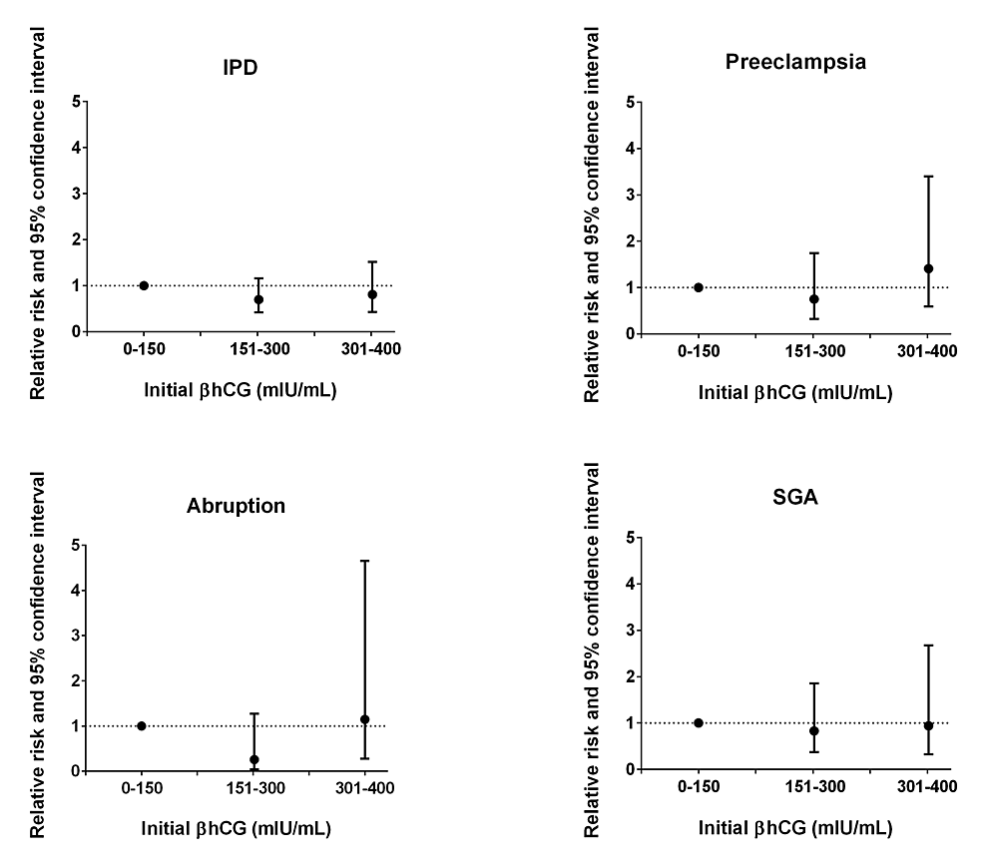
Adjusted risk ratios of ischemic placental disease and its components in first donor IVF singleton pregnancies by thresholds of initial βhCG Note: Data presented as risk ratio and 95% CI. Adjusted for maternal age, parity, and embryo age at time of initial βhCG with the exception of donor preeclampsia, which was adjusted for maternal age and parity due to convergence issues. IPD: ischemic placental disease; SGA: small for gestational age. 0-150 (n=61), 151-300 (n=103), 301-450 (n=38).

Similarly, there were no significant associations observed among donor IVF cycles (Figure 2).

**Figure 3:**
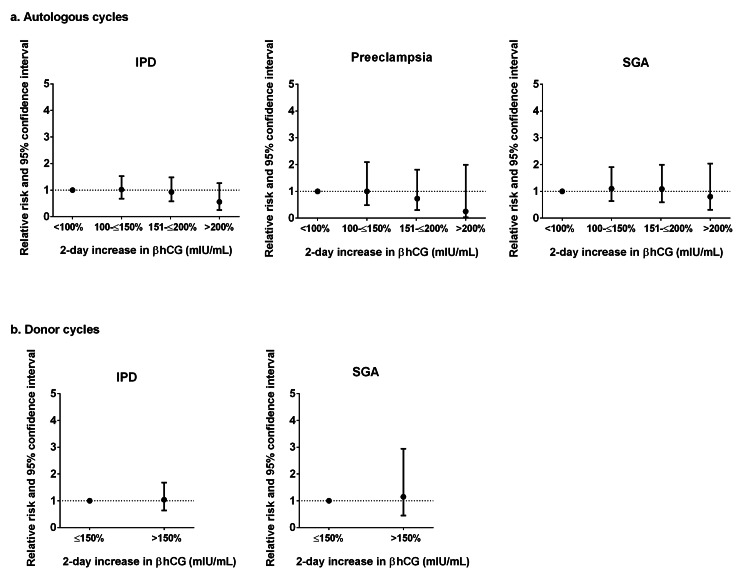
Adjusted risk ratios of ischemic placental disease and its components in singleton autologous (a) and donor (b) IVF pregnancies by two-day increases in βhCG Note: Data presented as risk ratio and 95% CI. Adjusted for maternal age and parity. IPD: ischemic placental disease; SGA: small for gestational age. Autologous: <100% (n=210), 100–≤150% (n=267), 150–≤200% (n=173), >200% (n=66). Donor: ≤150% (n=63), >150% (n=39). Adjusted models for autologous placental abruption and donor cycles for preeclampsia and placental abruption did not converge.

Though not significant, the results suggested a decreased risk of IPD, preeclampsia, and SGA with an increasing two-day percent increase in βhCG for patients among all autologous IVF cycles (Figure 3a). In contrast, the results suggested an increased risk among donor IVF cycles; however, the sample size was too small to compute adjusted RR for all of the IPD components (Figure 3b).

**Figure 4:**
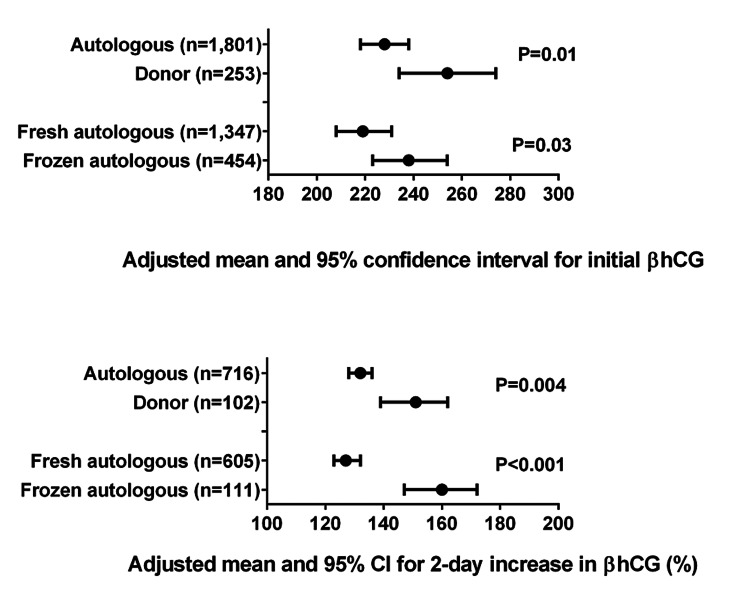
Initial and two-day percent increase in βhCG for singleton autologous and donor IVF patients Models for initial βhCG are adjusted for embryo age at the time of initial βhCG measurement and repeated deliveries from the same individual; models for two-day increase in βhCG are adjusted for repeated deliveries.

Frozen IVF cycles had a significantly higher mean initial βhCG value (P=0.03) compared to fresh cycles after accounting for embryo age at initial βhCG measurement and repeated deliveries from the same individual; frozen cycles also had a larger mean two-day percent increase in βhCG (P<.001) compared with fresh cycles after accounting for repeated deliveries (Figure 4). Similarly, donor IVF cycles had a significantly higher adjusted mean initial βhCG value (P=0.01) and a larger adjusted mean two-day percent increase in βhCG (P=0.004) compared with autologous IVF cycles.

## Discussion

We did not find any significant risk of IPD or its components associated with the initial or two-day percent increase in βhCG for both autologous and donor IVF cycles at our institution over a 19-year period. However, we observed statistically significant higher initial and two-day percent increases in βhCG when comparing autologous frozen with autologous fresh IVF and autologous with donor IVF. This highlights the difference in endometrial endocrine environments and immune tolerance.

Previous studies have reported on the use of βhCG in maternal serum aneuploidy screening [20,21] in combination with ultrasound nuchal translucency/crown rump length [22] or Dopplers [23,24] and have been correlated with placental abruption, intrauterine growth restriction (IUGR), low birth weight, preterm birth, SGA, and preeclampsia in natural conceptions. Previous findings have been inconsistent in that late first-trimester βhCG values in natural conceptions may only be mildly reduced or not significantly altered, while in the second trimester, βhCG values may be elevated in pregnancies with preeclampsia and IUGR [24]. Although prior studies have demonstrated that patients with low levels of βhCG on day 12 after a cleavage stage embryo transfer had an increased risk of preeclampsia [25], we did not observe this in our cohort. A prior study showed that the risk of IPD was not significantly different between frozen and fresh IVF cycles in singleton pregnancies [5]. While we did not find any significant associations with initial βhCG and the risk of IPD or its components, these findings contribute to the literature examining the utility of βhCG in pregnancy screening.

Morse et al. evaluated the rate of early βhCG rise and its association with adverse pregnancy outcomes after IVF. They concluded that a slower βhCG rise in fresh and frozen IVF cycles was associated with low birth weight and SGA but not with preterm birth or hypertensive disorders of pregnancy [9]. In our study, we examined two-day percent increases in βhCG as opposed to the rate of its rise, as a two-day percent increase is available in real-time and is already used clinically. However, our two-day median βhCG rise of 128% in autologous IVF pregnancies was consistent with previous reports [9,11].

When comparing fresh to frozen IVF cycles, the supraphysiologic endometrial environment creates an altered endocrine milieu [26,27]. With the higher estradiol levels during ovarian stimulation, this can impact the peri-implantation environment with changes in trophoblast differentiation affecting implantation, placental development, and perinatal outcomes [26,28]. Modulation of endometrial cell function with differential expression in over 200 endometrial genes in stimulated cycles versus natural cycles supports this hypothesis [26,28]. A previous study using these data concluded that the risk of IPD was decreased in frozen compared with fresh IVF cycles [5], which correlates with frozen cycles having a more "physiologic" environment that favors improved early placentation with a natural or artificial preparation [29]. Our findings of a higher initial βhCG in frozen compared with fresh IVF cycles are consistent with previous research [30]. Furthermore, two previous studies have shown that after the initial βhCG difference in fresh and frozen IVF cycles, the rate of rise was consistent [9,30].

Previous authors assessed first-trimester serum markers (βhCG and pregnancy-associated plasma protein-A) in donor and autologous IVF and found a similar multiple of the median between the two groups [31,32]. A previous study reported that initial βhCG was similar between the donor and autologous IVF cycles [33], though the overall sample size was small, and the number of donor patients was not clearly stated. In our donor population, we observed a higher initial and two-day percent increase in βhCG. This difference may stem from the different immunologic responses in donor oocyte patients [28]. Placental histology comparing donor and autologous IVF patients may provide some rationale for the differences we saw in our study [34]. Prior histologic studies have demonstrated that chronic deciduitis with fibrinoid deposition on the basal plate of the placenta is seen only in pregnancies resulting from oocyte donation [34]. This represents the site where extravillous cytotrophoblasts invade the maternal decidua, leading to potential increased syncytial knots causing decreased intervillous blood flow [34]. While this could be seen as a graft rejection versus host phenomenon, this may actually represent an immune effort to suppress rejection [34]. However, further research is needed to better understand βhCG levels and appropriate implantation in autologous and donor IVF cycles.

Our study's limitations include its retrospective design. Given the long study period, the influence of IVF clinical and laboratory practice changes could have influenced results. While these changes would be expected to affect all IVF cycle types equally, there were few frozen IVF cycles performed in the first half of the study period. We were unable to control for IVF stimulation protocols, frozen IVF cycle type, or use of intracytoplasmic sperm injection. Also, we did not have access to medical comorbidities such as pre-gestational diabetes, chronic hypertension, or smoking status. Lastly, these data were from a single academic institution, which limits its generalizability.

Our study has several notable strengths, which include a large data set with autologous (fresh and frozen) and donor IVF pregnancies. However, future studies need a larger sample size. Multiples were not included in this study due to the effect on early βhCG curves and perinatal outcomes. Any institutional obstetrical data not available was verified with the Massachusetts Department of Public Health. Internal validation was conducted, and approximately 90% of pregnancies with a diagnosis code for preeclampsia or placental abruption were confirmed by medical record review [8]. To the best of our knowledge, this is the first study to look at early βhCG in autologous and donor IVF and risks for IPD and its components. We included a composite outcome comprised of four perinatal outcomes that share a common risk factor and similar pathophysiology.

## Conclusions

The present study found no increased risk for IPD or its components when looking at the initial or two-day percent increase in βhCG for autologous and donor IVF. Significantly higher initial and two-day percent increases in βhCG were seen when comparing autologous frozen with autologous fresh IVF cycles and donor with autologous IVF cycles. Infertility patients are monitored closely with serum blood and ultrasound in early pregnancy and can serve as an ideal group to better understand early placentation and correlations with pregnancy outcomes. Further studies are needed to identify early markers in IVF that may predict adverse perinatal outcomes.
